# Reliable labels, uncertain meaning: Classifications for medial meniscus posterior root tears lack consensus and arthroscopy‐anchored accuracy

**DOI:** 10.1002/ksa.70130

**Published:** 2025-10-28

**Authors:** Michelangelo Palco, Gabriele Giuca, Filippo Familiari, Danilo Leonetti, Robert F. LaPrade, Roberto Simonetta

**Affiliations:** ^1^ Department of Orthopaedic and Trauma Surgery Casa di Cura Caminiti Villa San Giovanni Italy; ^2^ Section of Orthopaedics and Traumatology, Department of Biomedical Sciences and Morphological and Functional Images University of Messina Messina 98122 Italy; ^3^ Department of Orthopaedic and Trauma Surgery and Research Center on Muscoloskeletal Health Magna Graecia University Catanzaro Italy; ^4^ Twin Cities Orthopedics Edina Minnesota USA

**Keywords:** classification, magnetic‐resonance imaging, medial meniscus, posterior‐root tears, systematic review

## Abstract

**Purpose:**

Multiple, nonharmonised classification systems exist for posterior medial meniscus root tears (PMMRT), yet their diagnostic accuracy and observer reliability remain unclear. This PRISMA‐2020 systematic review aimed to catalogue available PMMRT classifications and to summarise accuracy and reliability metrics.

**Methods:**

Searches (PubMed, Scopus, Cochrane CENTRAL, Ovid MEDLINE/Embase; English; humans; no date/design limits; 9 September 2025) identified 2849 records; 1920 unique records were screened and 228 full texts assessed; six studies were eligible. Risk of bias followed QUADAS‐2 for diagnostic accuracy and GRRAS/COSMIN for reliability. Study‐specific interobserver *κ* and measurement ICC (with 95% confidence interval [CIs]) were extracted. Arthroscopy‐validated diagnostic accuracy was available from one study; no pooling was performed.

**Results:**

One arthroscopy‐validated MRI study reported high accuracy for radial PMMRT (sensitivity 0.90; specificity 0.94). Interobserver agreement for MRI‐based classification/diagnosis was high in two studies (*κ* = 0.86–0.93). MRI measurement repeatability for extrusion/tear‐gap was good‐to‐excellent (ICC: 0.80–0.92). No study prospectively linked subtype to treatment or outcomes.

**Conclusion:**

MRI‐based PMMRT classifications show high interobserver agreement, but arthroscopy‐validated accuracy evidence remains limited (single study); no system can be endorsed as most robust. A consensus, outcome‐anchored anatomical taxonomy and multicenter, arthroscopy‐validated studies linked to clinical outcomes are required before standardisation.

**Clinical Trial Registration:**

PROSPERO registration number: CRD420251103590.

**Level of Evidence:**

Level III.

**Abbreviations**
CI: confidence interval
COSMIN: consensus‐based standards for the selection of health measurement instruments
GRRAS: guidelines for reporting reliability and agreement studies
ICC: intraclass correlation coefficient
MRI: magnetic resonance imaging
PICO: population, index test, comparator, outcomes
PMMRT: posterior medial meniscus root tear
PRISMA: preferred reporting items for systematic reviews and meta analyses
QUADAS‐2: quality assessment of diagnostic accuracy studies 2
κ: Cohen's kappa

## INTRODUCTION

Posterior medial meniscus root tears (PMMRT) are radial or oblique disruptions within ~9–10 mm of the tibial insertion, or bony avulsions, that uncouple hoop‐stress transmission and elevate medial compartment loading [15]. Cadaveric models show increased peak contact pressure and reduced contact area after root avulsion, with repair partially restoring loading towards native values [7, 12, 15]. Finite‐element simulations corroborate impaired load sharing at the posterior root, although the magnitude of global tibiofemoral stress elevation varies with motion and boundary conditions [10, 16]. Longitudinal clinical series further suggest worse radiographic progression and arthroplasty conversion after nonrepair/meniscectomy compared with repair, with effect sizes differing by cohort and technique [4, 11].

Multiple classification constructs have been proposed across arthroscopy and MRI. Arthroscopy‐based morphology schemes (e.g., LaPrade) describe partial/complete radial tears within 9 mm, complex detachment and avulsion patterns [12], with later extensions addressing degenerative variants and tissue loss [5]. MRI‐oriented systems (e.g., Choi; gap‐based approaches) emphasise radial cleft/gap, stump displacement and extrusion on proton‐density fat‐suppressed sequences and Kim gap‐based scheme [9] and arthroscopy–MRI correlation frameworks describe displacement categories linked with extrusion [14]. Despite overlapping intent, terminology and operational criteria only partially align across systems; reporting conventions and reader training are heterogeneous, measurement standards are uneven, and prospective outcome anchoring is absent. Arthroscopy‐validated diagnostic accuracy specific to PMMRT on MRI is available from one study (radial posterior root) [13], whereas interobserver agreement has been reported in two MRI studies (*κ* ≈ 0.86–0.93) and measurement repeatability for gap/extrusion appears good‐to‐excellent where assessed [2, 9]. Recent MRI work highlights adjunctive signs and quantitative approaches, for example, flexion‐position cleft‐width and combined cleft + truncated‐triangle signs, together with extrusion‐centric indices as potentially standardizable markers [3, 8, 17].

Taken together, the literature depicts a plurality of partially overlapping constructs rather than a unified, clinically validated taxonomy. There is no universally accepted, outcome‐anchored anatomical classification for PMMRT at present, and evidence is insufficient to designate any system as ‘most robust’. This systematic review catalogues published PMMRT classification systems and summarises, at the study level, arthroscopy‐validated diagnostic accuracy, observer agreement (inter‐ and intraobserver *κ*), and measurement repeatability (ICC), explicitly identifying where heterogeneity and data scarcity preclude pooling or reliable between‐system comparisons.

It was hypothesised a priori that MRI‐oriented classifications would show high interobserver agreement (operationally *κ* ≥ 0.80) with similar intraobserver agreement when available; that arthroscopy‐validated accuracy data would be too sparse and heterogeneous to support pooling or nominate a single superior system; and that quantitative MRI measures relevant to PMMRT (e.g., extrusion, radial gap) would demonstrate good‐to‐excellent repeatability (ICC ≥ 0.80) where assessed [2, 9, 13].

## MATERIALS AND METHODS

### Protocol and registration

The review protocol (objectives, eligibility criteria, primary and secondary outcomes, analytic approach) was lodged with PROSPERO before database searching. No deviations occurred from the registered protocol.

### Eligibility criteria

Studies were eligible if they enrolled adults or late adolescents (≥18 years) with a medial meniscus posterior‑root tear (PMMRT) confirmed by arthroscopy or by magnetic‑resonance imaging. Studies were excluded if assessed detection of PMMRT without applying a defined classification framework with operational categories. Arthroscopic inspection served as the preferred reference standard; when this was unavailable, the study had to specify an expert musculoskeletal‑radiologist MRI consensus as a surrogate reference. Eligible reports provided either (i) 2 × 2 diagnostic‑accuracy data, true‑positive, false‑positive, false‑negative and true‑negative counts, from which sensitivity and specificity could be calculated, or (ii) reproducibility statistics such as Cohen's *κ*, weighted *κ*, or intraclass correlation coefficients. Randomised or nonrandomised clinical investigations, prospective or retrospective cohort studies, cross‑sectional diagnostic‑accuracy assessments and technical notes were included. Single‑case or small case‑series reports involving fewer than 10 knees, purely cadaveric biomechanical experiments and narrative or systematic reviews were excluded.

### Information sources and search strategy

Four sources were systematically searched on 9 September 2025: PubMed (NCBI), Scopus (Elsevier), Cochrane CENTRAL (The Cochrane Library) and Ovid multidatabase (MEDLINE and Embase). No date limits or study‐design filters were applied; language was restricted to English; humans only. The exact, database‐specific queries were:

PubMed (NCBI):

(‘medial meniscus’[tiab] OR ‘Meniscus, Medial’[Mesh]) AND ((‘root tear’[tiab] OR ‘root avulsion’[tiab] OR ‘root injury’[tiab] OR ‘posterior root’[tiab] OR ‘posterior horn root’[tiab] OR MMPRT[tiab] OR PMMRT[tiab])) AND (classification[tiab] OR grading[tiab] OR subtype[tiab] OR type[tiab] OR taxonomy[tiab] OR categor*[tiab] OR pattern*[tiab])

Scopus (TITLE‐ABS‐KEY):

TITLE‐ABS‐KEY(‘medial meniscus’) AND TITLE‐ABS‐KEY(‘root tear’ OR ‘root avulsion’ OR ‘root injury’) AND TITLE‐ABS‐KEY(classification OR grading OR subtype)

Cochrane CENTRAL (ti,ab,kw):

(medial meniscus):ti,ab,kw AND (‘root tear’ OR ‘root avulsion’ OR ‘root injury’):ti,ab,kw AND (classification OR grading OR subtype):ti,ab,kw

Ovid (MEDLINE + Embase; field tags.ti,ab.):

medial meniscus.ti,ab.; (root tear OR root avulsion OR root injury).ti,ab.; (classification OR grading OR subtype).ti,ab.; 1 AND 2 AND 3

The searches returned 1091 records in PubMed, 1469 in Scopus, 8 in CENTRAL and 281 in Ovid (prededuplication counts). Grey‐literature sources included *OpenGrey*, and major orthopaedic‐congress abstract books (AAOS, ESSKA, ISAKOS, AOSSM 2015–2025). Reference lists of all eligible full texts were hand‐screened.

### Study selection

All records were exported (RIS/CSV), merged, and exported to EndNote X20 and deduplicated electronically then manually. Two reviewers (G.G. and M.P.) screened titles and abstracts independently. Cohen's *κ* for title/abstract agreement was 0.82, indicating near‐perfect concordance. Full‐text review followed identical independent appraisal; disagreements (*n* = 6) were resolved through discussion with a third reviewer (F.F.). The PRISMA 2020 flow diagram reports database‐level yields, duplicates removed, and study counts by screening phase (Figure 1).

**Figure 1:**
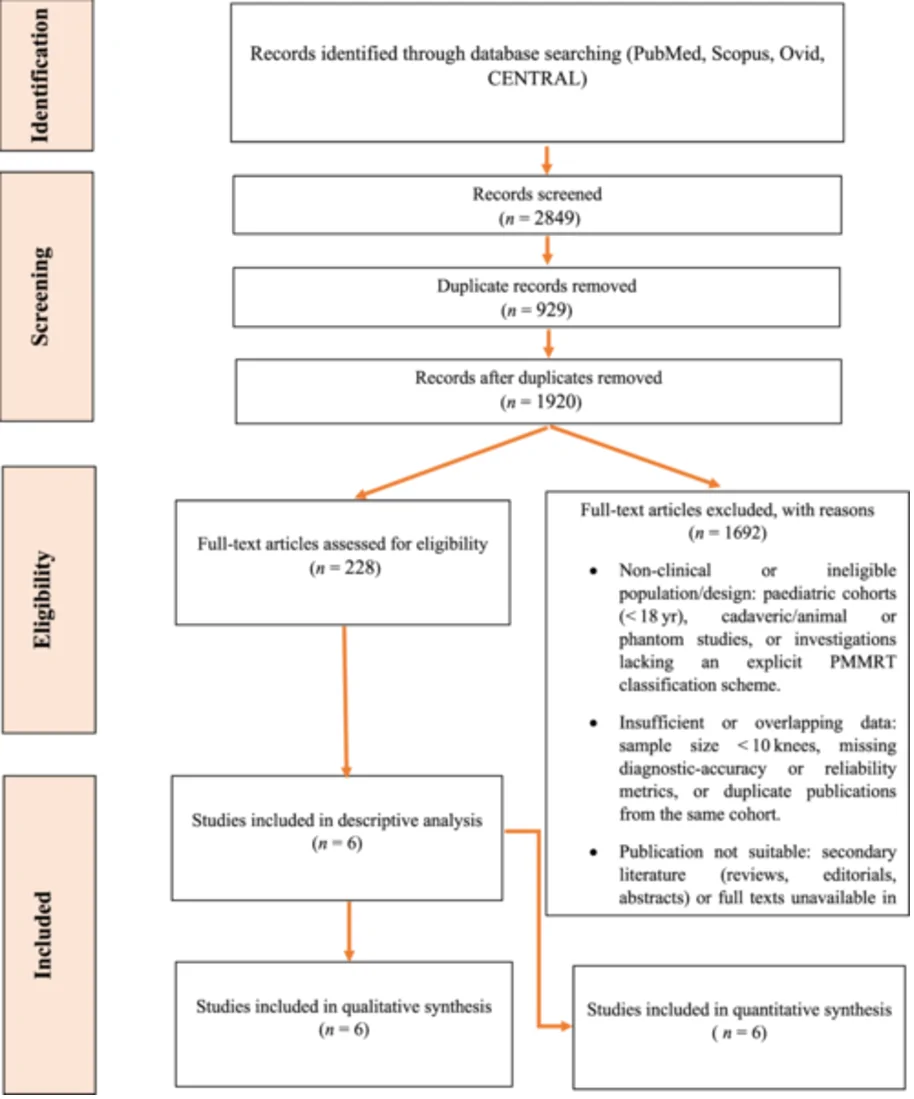
Preferred reporting items for systematic reviews and meta analyses (PRISMA) flow diagram of study selection.

### Data‐collection process

All extracted information was independently crosschecked against the original articles before consensus entry into the master spreadsheet. Data fields were grouped into six domains. First, the bibliographic profile of the study was documented, including the author, publication year, country, institutional setting, funding source, study design and Oxford level of evidence. Second, patient demographics and imaging particulars were captured, including age, sex distribution, body mass index, symptom chronicity, MRI field strength and pulse sequences, and where applicable, the arthroscopic technique. Third, all elements of the index classification system were detailed: its name, date of first description, total number of categories, objective defining criteria (e.g., radial‐gap width or meniscal extrusion), and its modality of application (MRI, arthroscopy, or both). The fourth domain comprised diagnostic‑accuracy metrics: true‑positive, false‑positive, false‑negative and true‑negative counts, together with any sensitivity, specificity, positive‑predictive value and negative‑predictive value reported by the authors. Fifth, reproducibility metrics were documented, including the number and experience of the readers, blinding procedures, and inter‐ or intraobserver agreement statistics (*κ* or ICC values) with corresponding 95% confidence intervals (CIs).

### Risk‐of‐bias assessment

Diagnostic accuracy was appraised with QUADAS‐2; agreement/reliability reporting followed GRRAS and the COSMIN checklist.

### Effect measures and synthesis methods

Study‐level sensitivity and specificity were calculated with Wilson score 95% CIs. Because only one study reported PMMRT‐specific 2 × 2 data versus arthroscopy, diagnostic accuracy was summarised at the single‐study level; no formal meta‐analysis was performed. Study‐specific *κ* and ICC with 95% CIs were extracted; no ICC → *κ* conversions were performed. Intraobserver *κ* and measurement repeatability (ICC), when available, were summarised narratively. Computations used R 4.4 and Python 3.11; figures, Matplotlib v3.8.

## RESULTS

### Search results

The search identified 2849 records across PubMed, Scopus, Cochrane CENTRAL and Ovid (MEDLINE + Embase). After removal of 929 duplicates, 1920 unique records were screened; 228 full texts were assessed; six studies met eligibility (Figure 1).

### Study characteristics

The included studies evaluated PMMRT classification constructs derived from arthroscopy‐based schemas (e.g., radial/oblique tear within ~1 cm of the tibial insertion; bony avulsion) and MRI‐oriented descriptors (e.g., radial‐gap/cleft, truncated‐triangle/extrusion context). Imaging protocols and reader expertise were heterogeneous (Table 1). A cross‐study map of endpoint reporting (diagnostic accuracy; inter‐ and intraobserver *κ*; measurement ICC) is provided in Figure 2.

**Table 1:** Summarises the six included investigations (*n* = 944 knees).

Reference	Study design	Evidence level	Classification basis	Sample size (knees)	Mean age (year)	Classification system	Interobserver reliability	Diagnostic accuracy (sensitivity/specificity)
[12]	Case series	IV	Arthroscopy	71	32.9	5‐type morphology	NR	NR
[2]	Retrospective cohort	III	Magnetic resonance imaging (MRI)	419	45.7	Posterior medial meniscus root tear (PMMRL) (3 grades)	*κ* 0.86 / 0.93	NR
[9]	Prospective cohort	III	Arthroscopy + MRI	116	58.6	5‐type tear‐gap	Intraclass correlation coefficient 0.89–0.92	NR (measurement reliability reported)
[6]	Technical note	V	Arthroscopy	—	—	Extended LaPrade (Types 6–8)	NR	NR
[14]	Case control	III	Magnetic resonance imaging (MRI) + Arthroscopy	146	55.2	General knee MRI at 0.25 T	NR	NR
[13]	Retrospective cohort	III	MRI	192	39.7	Radial‐tear detection	*κ* 0.93	0.90 / 0.94

**Figure 2:**
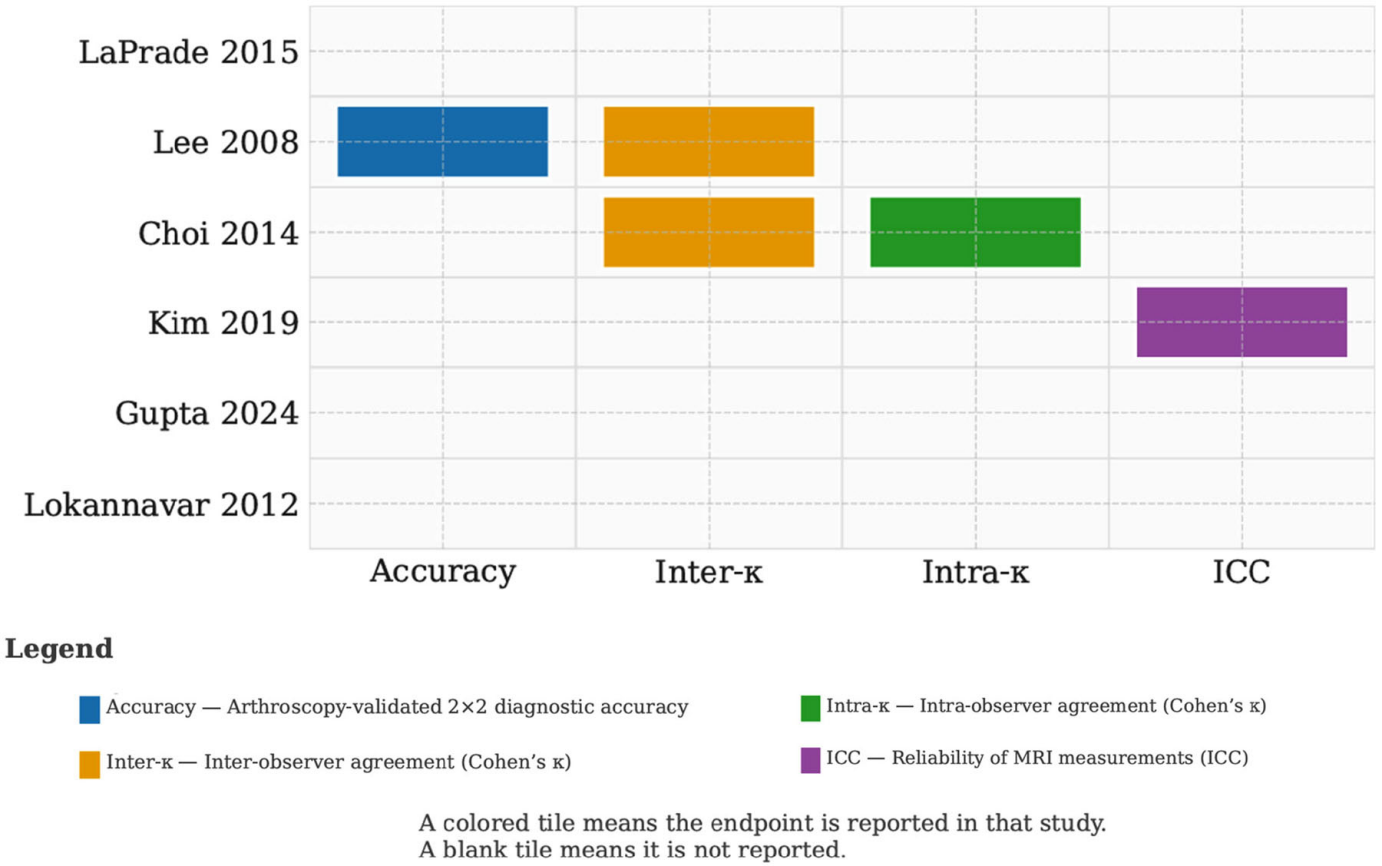
Evidence map of endpoint reporting across posterior medial meniscus root tear (PMMRT) classification studies. Columns: Accuracy (arthroscopy‐validated 2 × 2), Inter‐*κ*, Intra‐*κ*, intraclass correlation coefficient (ICC); coloured tiles = reported, blank = not reported.

### Diagnostic accuracy

Arthroscopy‐validated MRI accuracy was available from one study, focused on radial PMMRT. Reported performance was high (sensitivity 0.90; specificity 0.94). No head‐to‐head accuracy comparisons between classification systems were feasible, and no pooling was undertaken due to the single arthroscopy‐validated data set (Figure 3).

**Figure 3:**
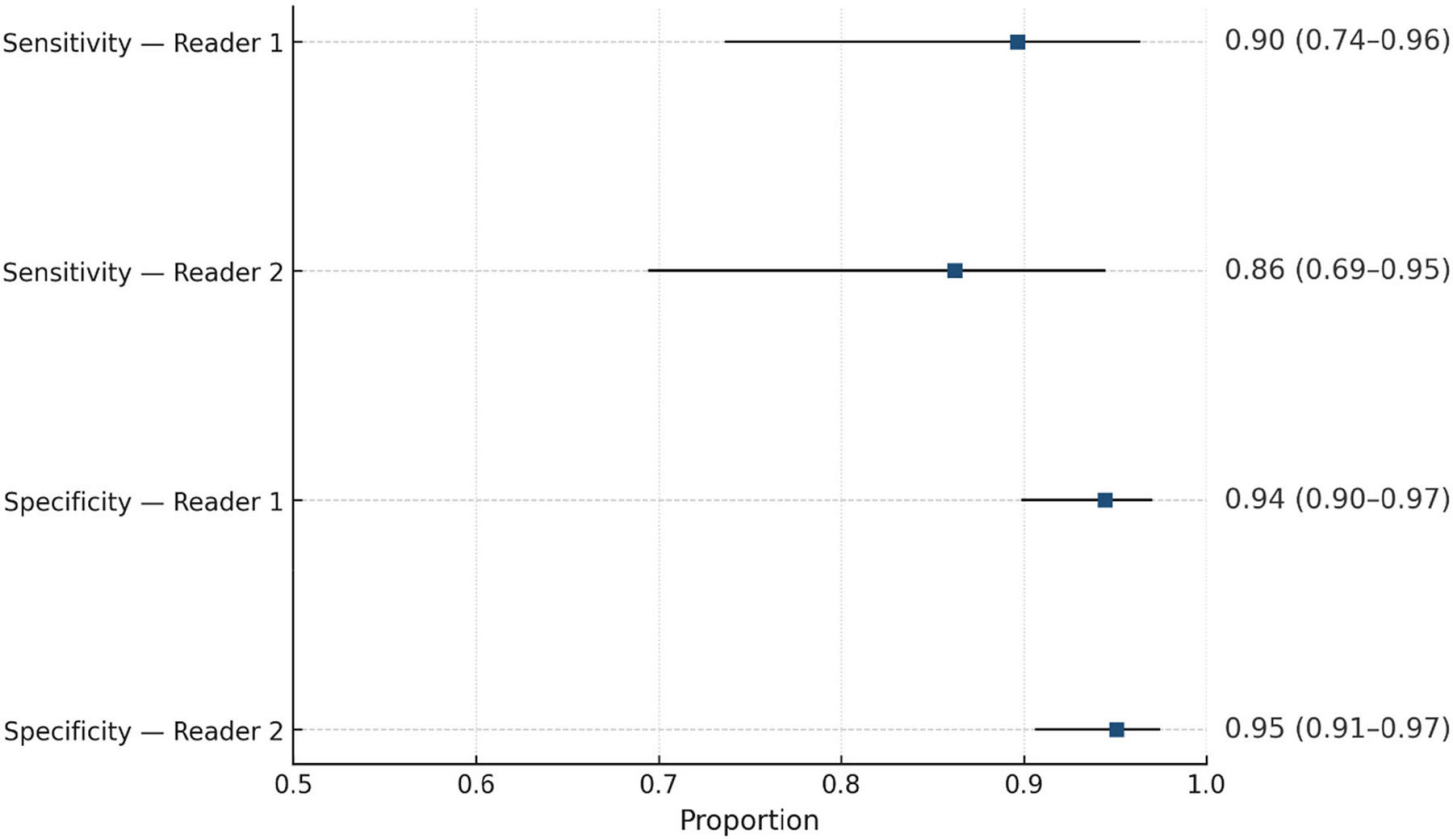
Sensitivity and specificity (with Wilson 95% confidence intervals) for magnetic resonance imaging (MRI) diagnosis of radial posterior medial meniscal root tears versus arthroscopy in Lee 2008, shown separately for two independent readers.

### Observer reliability

Interobserver agreement for MRI‐based PMMRT classification/diagnosis was high in two studies (*κ* 0.86–0.93). Where reported, intraobserver agreement was high (e.g., *κ* ≈ 0.93). Given the small number of studies, no pooled *κ* is presented; study‐specific estimates and 95% CIs are shown in Figure 4.

**Figure 4:**
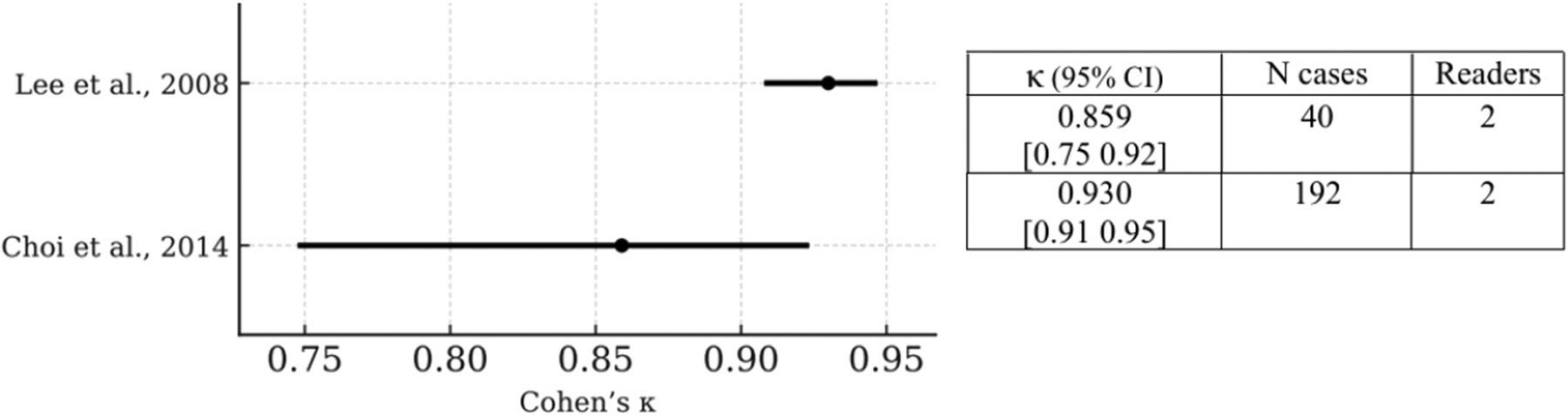
Interobserver agreement (Cohen's *κ*) for magnetic resonance imaging (MRI) diagnosis/classification of posterior medial meniscus root tear (PMMRT). Points show *κ* with 95% confidence intervals (CIs); the right panel reports *κ* (95% CI), number of cases (*N*), and readers.

### Measurement repeatability (ICC)

MRI‐derived quantitative measures pertinent to PMMRT (e.g., extrusion, tear‐gap width) demonstrated good‐to‐excellent repeatability where reported (ICC ≈ 0.80–0.92). Detailed metrics and readers/cases per study are provided in Table 1.

### Risk of bias and applicability

Risk‐of‐bias assessment (QUADAS‐2 for diagnostic accuracy; GRRAS/COSMIN for reliability) indicated variable quality across domains, with common concerns in patient selection, reference‐standard implementation (arthroscopy not universal) and small sample sizes; applicability concerns were low‐to‐moderate overall.

## DISCUSSION

Rather than a single, anatomically grounded standard, current PMMRT literature comprises partially overlapping arthroscopy‐ and MRI‐based constructs with inconsistent operational criteria and scarce validation; as such, no consensus, outcome‐anchored anatomical taxonomy is available for clinical adoption.

### Comparison with existing literature

Arthroscopy‐validated MRI accuracy for radial PMMRT was high in a single study. Cross‐entity comparisons (e.g., ramp lesions with reported generic MRI detection around 50%) are indirect and should be interpreted cautiously given differences in lesion biology, reference standards and imaging protocols [1]. Overall, current literature reflects partially overlapping classification constructs rather than a unified, outcome‐anchored anatomical taxonomy suitable for routine clinical adoption. MRI‐based frameworks using explicit criteria (e.g., Choi et al. [2] Kim et al. [9]) demonstrated higher observer agreement than morphology‐only descriptions. Beyond tear‐morphology taxonomies, extrusion‐based constructs may indirectly stratify lesion severity. The Meniscal Extrusion Index (MEI) classification demonstrated reliability and potential biomechanical relevance across diverse meniscal lesions and could enrich future PMMRT frameworks by incorporating percentage extrusion as a standardised displacement marker [3].

### Clinical implications

For the practicing knee surgeon, classification systems should not only describe morphology but also guide management. Recognising a complete radial tear close to the tibial insertion or a type 5 avulsion is clinically relevant, as these lesions are typically biomechanically unstable and may warrant transtibial pull‐out repair, often combined with medial collateral ligament release to reduce extrusion. Conversely, degenerative or partial tears with limited displacement in older, low‐demand patients may be treated nonoperatively or with meniscectomy, given the limited benefit of repair in advanced chondropathy. MRI‐based systems with high interobserver agreement, such as Choi's or Kim's gap‐based approach, offer reproducible preoperative information that can support surgical planning. However, the lack of a universally validated taxonomy forces surgeons to rely on personal interpretation or hybrid descriptors, which reduces comparability of outcomes and complicates communication in multidisciplinary teams. Establishing a consensus classification, validated against both arthroscopy and long‐term patient outcomes, is therefore essential to translate imaging findings into standardised surgical algorithms and to optimise decision‐making in routine practice.

### Methodological considerations

Our synthesis was deliberately constrained to what the evidence could support. For diagnostic accuracy, only one included study provided arthroscopy‑validated 2 × 2 data for MRI diagnosis of radial posterior medial meniscal root tears; consequently, accuracy was summarised at the single‑study level and no bivariate/HSROC or between‑study heterogeneity estimates were attempted. Consequently, subgroup hypothesis testing, evaluations of small‐study effects and meta‐regression for accuracy were avoided. Regarding observer agreement, categorical agreement (*κ*) and measurement reliability (ICC) were analysed separately. Any conversion of ICC to *κ* was expressly avoided. Finally, *κ* estimates for MRI‐based diagnosis and classification were reported alongside ICC values for MRI‐derived continuous measures (specifically, absolute extrusion, relative extrusion and tear‐gap width). Frameworks that did not furnish operational categories or PMMRT‑specific diagnostic validation (e.g., general low‑field knee MRI cohorts and technical notes without patient‑level testing) were not used in quantitative accuracy synthesis and were treated narratively to avoid scope drift from classification to mere detection. Across studies, important design features, reader experience and blinding, MRI protocols and field strengths, sequence/plane availability and the choice of reference standard, were heterogeneous and, in several instances, incompletely reported; these factors limit comparability and may introduce spectrum and verification biases. Finally, given the limited number of eligible studies, transparency and reproducibility were prioritised over statistical ambition. This was operationalized by a priori specification of transformations and random‐effects estimators appropriate to each metric, comprehensive documentation of all exclusions, and the provision of study‐level data extractions. This framework ensures that future prospectively designed, multicenter validations, which utilise harmonised operational criteria, blinded readers and a uniform arthroscopic reference standard, can be incorporated without requiring a reconstruction of the analytical framework. Overall, the evidence base is highly limited: only six studies met inclusion, and few reported both accuracy and reliability within the same framework. Consequently, we cannot endorse any single system as ‘most robust’ at present.

### Future research directions

A four‐step agenda is therefore advocated: (1) achieving Delphi consensus among international orthopaedic knee societies to merge existing systems into a single hierarchical classification encompassing tear morphology, displacement and meniscal extrusion; (2) conducting prospective multicenter validation with blinded MRI readers and arthroscopic reference standards, reporting both accuracy and reliability metrics; (3) implementing prospective cohort studies to correlate classification subtypes with surgical repair techniques (e.g., transtibial tunnels versus suture anchors) and 2‐year patient‐reported outcomes; and (4) developing artificial intelligence tools through training convolutional neural networks on labelled MRI datasets to automate subtype assignment, thereby reducing interreader variability and enabling large‐scale registry studies.

### Limitations

The evidence base is small and heterogeneous (six studies; variable endpoints), precluding pooled diagnostic‐accuracy estimates and robust between‐system comparisons. Only one study provided PMMRT‐specific 2 × 2 data versus arthroscopy; hence sensitivity/specificity were summarised at the single‐study level. Reporting of specificity and reference standards was inconsistent; spectrum and verification biases are possible. Clinical outcomes (e.g., International Knee Documentation Committee scores, radiographic OA progression) were beyond our remit and remain essential for establishing prognostic validity.

## CONCLUSION

MRI‐based PMMRT classifications demonstrate high interobserver agreement in two studies, but no universally accepted, outcome‐anchored anatomical classification exists; arthroscopy‐validated diagnostic accuracy is limited to a single study and prospective outcome linkage is absent. No classification can presently be endorsed as ‘most robust’; consensus taxonomy and multicenter validation against arthroscopy and clinical outcomes are required.

## AUTHOR CONTRIBUTIONS

*Conceived and designed the paper*: Michelangelo Palco and Gabriele Giuca. *Collected the data*: Gabriele Giuca, Filippo Familiari and Danilo Leonetti. *Contributed data or analysis tools*: Filippo Familiari and Roberto Simonetta. *Wrote the paper*: Gabriele Giuca and Michelangelo Palco. Robert F. LaPrade reviewed the paper.

## CONFLICT OF INTEREST STATEMENT

The authors declare no conflicts of interest.

## ETHICS STATEMENT

Not applicable for systematic reviews based on published data; no IRB approval required.

## Supporting information


**Table S1** Overview of PMMRT classification systems.

## Data Availability

This is a PRISMA‐2020 compliant systematic review/meta‐analysis based on published studies. The study selection/extraction files and analytic code (R 4.4/Python 3.11) can be shared on reasonable request to the corresponding author; all data synthesised derived from cited sources.
