# Supplementary material for: Reliable labels, uncertain meaning: Classifications for medial meniscus posterior root tears lack consensus and arthroscopy‐anchored accuracy
Source: Knee Surg Sports Traumatol Arthrosc. 2025 Oct 28;34(11):3908–16. doi: 10.1002/ksa.70130 (PMC13703782; doi:10.1002/ksa.70130)
Supplement: Supplementary file 1 [file KSA-34-3908-s001.docx]

**Table S1:** Overview of PMMRT classification systems.

| **Author** | **Modality** | **Core concept / Grades** | **Reported validation** | **Notes / Typical use** |
| --- | --- | --- | --- | --- |
| LaPrade CM  (2015) [12] | Arthroscopy | 5 morphology types (partial; radial within 9 mm; bucket‑handle w/ detachment; complex w/ detachment; avulsion) | Widely cited morphological taxonomy; no standardized displacement quantification | Historical anchor; descriptive intra‑op classification |
| Gupta S (2023)  [6] | Arthroscopy | Extended LaPrade (types 6–8). Adds horizontal, meniscocapsular separation, >10 mm tissue loss (Types 6–8) | Narrative/consensus extension; limited quantitative testing reported | Expands morphological breadth |
| Lee SY (2008)  [13] | MRI (arthroscopy‑validated) | Detection/classification of radial posterior root tears on PD‑FS sequences | Arthroscopy‑validated diagnostic accuracy reported for radial PMMRTs | Diagnostic reference for MRI accuracy against arthroscopy |
| Choi JY (2014)  [2] | MRI | 3‑grade scheme (degenerative, partial, complete) using PD‑FS criteria | High interobserver agreement reported (κ), intraobserver where available | Simple MRI‑oriented taxonomy |
| Kim JY (2019)  [9] | MRI / Hybrid metrics | 5 grades incorporating radial gap width on coronal MRI; extrusion/tear‑gap measurement | Good‑to‑excellent measurement repeatability (ICC) reported for gap/extrusion metrics | Bridges morphology with quantitative MRI metrics |
| Lokannavar HS (2012) [14] | Arthroscopy (w/ MRI correlation) | Stump displacement categories; correlation with MRI extrusion | Correlative evidence; limited reliability reporting | Links intra‑op findings to MRI displacement features |
